# Summative content analysis of the recommendations from Project ECHO Ontario Autism

**DOI:** 10.3389/fresc.2023.1096314

**Published:** 2023-03-30

**Authors:** Alanna Jane, Lisa Kanigsberg, Anmol Patel, Salina Eldon, Evdokia Anagnostou, Jessica Brian, Melanie Penner

**Affiliations:** ^1^ School of Medicine, Queen’s University, Kingston, ON, Canada; ^2^ Autism Research Centre, Holland Bloorview Kids Rehabilitation Hospital, Toronto, ON, Canada; ^3^ Department of Paediatrics, University of Toronto, Toronto, ON, Canada

**Keywords:** Autism, ECHO, diagnosis, summative analysis, medical education, health services

## Abstract

**Background:**

Practitioners report a lack of knowledge and confidence in treating autistic children, resulting in unmet healthcare needs. The Extension of Community Healthcare Outcomes (ECHO) Autism model addresses this through discussion of participant-generated cases, helping physicians provide best-practice care through co-created recommendations. Recommendations stemming from ECHO cases have yet to be characterized and may help guide the future care of autistic children. Our objective was to characterize and categorize case discussion recommendations from Project ECHO Ontario Autism to better identify gaps in clinician knowledge.

**Methods:**

We conducted a summative content analysis of all ECHO Ontario Autism case recommendations to identify categories of recommendations and their frequencies. Two researchers independently coded recommendations from five ECHO cases to develop the coding guide. They then each independently coded all remaining cases and recommendations from three cycles of ECHO held between October 2018 to July 2021, meeting regularly with the ECHO lead to consolidate the codes. A recommendation could be identified with more than one code if it pertained to multiple aspects of autism care. Categories from the various codes were identified and the frequency of each code was calculated.

**Results:**

Of the 422 recommendations stemming from 62 cases, we identified 55 codes across ten broad categories. Categories included accessing community resources (*n *= 224), referrals to allied health and other providers (*n *= 202), ongoing autism care (*n *= 169), co-occurring mental and physical health conditions (*n *= 168), resources and tools for further learning (*n *= 153), physician to provide education and coaching to families (*n *= 150), promoting parent and family wellness (*n *= 104), supporting community autism diagnosis (*n *= 97), promoting patient empowerment and autonomy (*n *= 87), and COVID-19 (*n *= 26).

**Conclusion:**

This is the first time that recommendations from ECHO Autism have been characterized and grouped into categories. Our results show that advice for autism identification and management spans many different facets of community-based care. Specific attention should be paid to providing continued access to education about autism, streamlining referrals to allied health providers, and a greater focus on patient- and family-centered care. Physicians should have continued access to autism education to help fill knowledge gaps and to facilitate families' service navigation.

## Introduction

Autism is a neurodevelopmental condition affecting one in 66 children and youth aged five to 17 in Canada (1). The complex nature of autism can lead to a difficult journey in the healthcare system for families. This includes unmet needs for healthcare and family supports, delayed or foregone care, and difficulty obtaining access to medical care and allied health services (2). Data from the United States National Survey of Children's Health showed that autistic children have a four times higher risk of unmet healthcare needs compared to children without disabilities (3). A survey collecting data from caregivers of autistic children found that around one third experienced unmet healthcare service needs like speech-language therapy, occupational therapy, and social skills training, while almost one quarter expressed needs for family support services such as respite care and parent/sibling support groups (4). Unmet healthcare needs may also extend to co-occurring mental health and physical health conditions, including dental health (5). Co-occurring mental health conditions in autistic individuals have been reported at an increased prevalence compared to the general population, including attention-deficit hyperactivity disorder (28%), anxiety disorders (20%), sleep-wake disorders (13%), disruptive, impulse-control, and conduct disorders (12%), and depressive disorders (11%) (6).

In the Canadian context, community practitioners caring for autistic individuals may include primary care physicians, consultant pediatricians, subspecialists, nurse practitioners, and other allied health providers situated outside of tertiary care centres (7). While primary care physicians have a wide scope of practice, which includes health promotion, preventative care, and the diagnosis and treatment of a variety of illnesses and injuries, consultant pediatricians are consulted by a child's primary care physician if there are concerns that require more specialized care (8). If the consultant pediatrician does not feel that they can provide the necessary expertise, they may refer to a subspecialist, such as a developmental pediatrician or multidisciplinary team. Community practitioners have reported a lack of knowledge, competence, and comfort in treating autistic children, contributing to these unmet healthcare needs (9, 10). Factors associated with discomfort in autism screening and diagnosing children include time limitations, a lack of familiarity with screening tools, and perceived difficulty of use (11, 12). Parents are reported to consult an average of three to five professionals before obtaining an autism diagnosis, with the time from first concern to diagnosis ranging from 12 to 55 months (13). Importantly, early diagnosis and therapies are associated with improvements in cognition, language, daily living skills, and social behaviour (14). The Canadian Paediatric Society (CPS) has stressed the importance of conferring a diagnosis at the earliest age possible, and highlights the need to expand autism diagnostic capacity by including community practitioners (15). Beyond diagnosis, the clinical complexity of autism care and associated co-occurring conditions may be difficult to address in the community, contributing to higher rates and durations of hospitalizations, greater expenditures, and greater use of psychotropic medications (16–20). There is a critical need to improve community-based autism care in early identification, diagnosis, and management of common issues such as sleep, constipation, and co-occurring neurodevelopmental and mental health conditions (16).

To address these shortcomings, targeted autism training programs may effectively educate community-based professionals on diagnosis and management. One widely used training program is the Extension for Community Healthcare Outcomes (ECHO) model, which aims to reduce barriers between specialists and community providers in treating complex conditions. ECHO uses videoconferencing to create learning communities through didactic learning, mentoring, and participant-generated case presentations and discussions. The ECHO model was first developed in 2003 by Dr. Sanjeev Arora, with its original application used in improving treatment outcomes for patients with hepatitis C virus (21).

ECHO Ontario Autism is a specific learning program under the ECHO Autism umbrella. When Project ECHO Ontario Autism was first developed, the curriculum from the original ECHO Autism pilot, designed in the United States, was expanded on and adapted to include the diagnosis of autism. This adaptation was made to meet the diagnostic needs in the Ontario community and increase the capacity of community practitioners to provide autism diagnoses. This led to the creation of two distinct learning objectives of Project ECHO Ontario Autism, in consultation with the Ontario Ministry of Health. Firstly, the program aims to build province-wide community capacity and ability to screen, diagnose, and manage children and youth with autism in Ontario (22). Secondly, the programs hopes to reduce wait times for specialized care by increasing community provider capacity, thereby reserving specialized care for complex cases (22). Furthermore, the team realized an important voice of autistic representation was needed, and prioritized the inclusion of autistic advocates to offer the team and participants a valuable lived experience perspective of the diagnostic process and navigating the system. While recent iterations of ECHO Autism programs have included elements of diagnosis and inclusion of autistic advocates, including ECHO Autism STAT and ECHO Autism Transition, respectively, ECHO Ontario Autism combines these factors while placing them in the contextual needs of the Ontario healthcare system (23, 24). In each session, a participant presents a challenging case of one of their patients to other participating community providers and an interdisciplinary “Hub” team composed of autistic advocates, parents of autistic children, and interdisciplinary autism specialists. Community participants and the Hub team discuss the case and co-develop recommendations for best-practice care, which are summarized by facilitators and disseminated to participants after each session.

Studies of the ECHO Autism program with primary care providers in Missouri found significant improvements in clinicians’ relationships with their patients and families, increased rates of accepting autism referrals for diagnostic evaluation, an increase in their autism caseload, increased self-efficacy scores from pre- to post-ECHO training, greater use of autism-specific resources, and higher participant-reported satisfaction (23, 25). A multi-centre North American trial demonstrated improved clinician knowledge and self-efficacy, although no changes were seen in autism screening or management of co-occurring conditions (26). A more recent study found that ECHO Autism facilitated successful integration of expertise in a remote and COVID-19-friendly fashion to improve knowledge and clinical practice (27). Reflective qualitative analyses of learning experiences of ECHO Autism participants in India and Hub facilitators in the United States have produced helpful markers of program evaluation and strengths (28, 29).

Despite existing studies, there remain important aspects of ECHO Autism that have not been explored. ECHO Autism case recommendations have not yet undergone qualitative or summative study; such examination is important to identify ongoing gaps in autism knowledge and teaching, with implications across medical autism training. Each ECHO session generates a unique list of recommendations, which provides an important data set for learning about post-training education and support needs for community-based clinicians. Existing qualitative evaluations of the ECHO learning model have focused on more traditional sources of information such as interviews and focus groups with ECHO participants, but no known studies have examined ECHO recommendations (30). As the data presented in our study are derived directly from the ECHO program and sessions, rather than participant reflections, our data set identifies timely on-the-ground care needs for autistic individuals and comprises a valuable dataset representing real-world challenges that clinicians are actively facing to best understand and support these care needs. The objective of our study was to characterize and categorize recommendations from Project ECHO Autism Ontario case discussions.

## Materials and methods

### Project ECHO Ontario Autism

**Figure 1:**
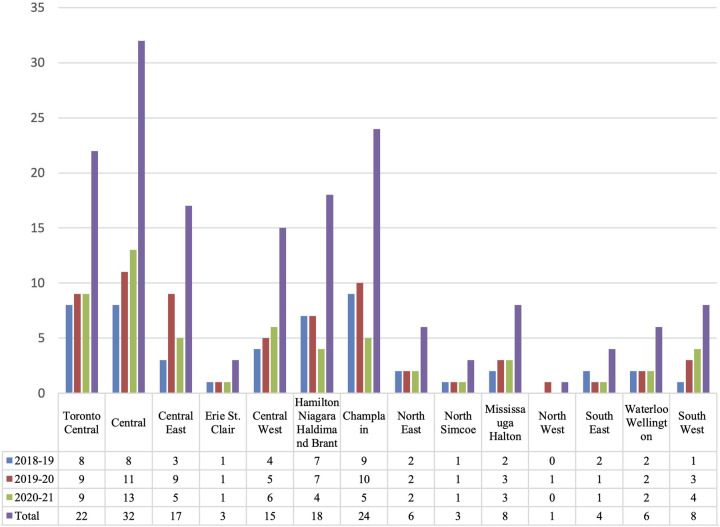
ECHO Ontario Autism participants by LHIN (*n*). Demographic information of ECHO Autism participants across all three cycles (2018–2019, 2019–2020, 2020–2021) by number of participants (*n*) from each Local Health Integration Network, including: (1) Toronto Central (*n* = 22), (2) Central (*n* = 32), (3) Central East (*n* = 17), (4) Erie/St. Clair (*n* = 3), (5) Central West (*n* = 15), (6) Hamilton/Niagara/Haldimand/Brant (*n* = 18), (7) Champlain (*n* = 24), (8) North East (*n* = 6), (9) North Simcoe (*n* = 3), (10) Mississauga/Halton (*n* = 8), (11) North West (*n* = 1), (12) South East (*n* = 4), (13) Waterloo/Wellington (*n* = 6), and (14) South West (*n* = 8).

ECHO Autism is currently offered through Holland Bloorview Kids Rehabilitation Hospital and funded by the Ontario Ministry of Health. At the time of writing this manuscript, three cycles of ECHO were completed from October 2018 to July 2021. Participation was steady across all three cycles, with 53, 51, and 43 attendees in at least one session in cycles 1, 2, and 3, respectively. ECHO Ontario Autism participation was open to any physician or nurse practitioner in Ontario, Canada. Information about the ECHO program was distributed through the Pediatric Section of the Ontario Medical Association. Demographic data about the professional disciplines of participants across all three cycles are presented in Table 1. Participants joined from across Ontario, with most located in the Greater Toronto Area (GTA). Demographic data about the geographic location of Ontario participants, organized by Local Health Integration Network, are presented in Figure 1. There were also a select few observers from other provinces and countries including Saskatchewan, Québec, British Columbia, The Bahamas, and India. Based on our inclusion criteria of participants working as physicians or nurse practitioners, we did not collect additional information about socioeconomic status and educational attainment for ECHO participants due to the homogenous and high level of education of our cohort.

**Table 1:** Professional disciplines of ECHO Ontario Autism participants.

Professional discipline	Participant number (*n*)
Pediatrician	84
Family Physician	10
Developmental Pediatrician	5
Nurse Practitioner	4
Neurologist	2

Participant numbers (*n*) across all three cycles of ECHO Ontario Autism.

Research ethics approval was obtained from the Holland Bloorview Research Ethics Board (REB #: 0267). All ECHO Autism case presentations and resulting recommendations were presented without any personal identifying information to ensure confidentiality. The recommendations were generated by the ECHO Autism team for distribution to ECHO participants; for this reason, we did not seek consent from the ECHO participants.

### Description of data set

Each ECHO session consists of 1–2 case presentations (cycle 1: 24 cases, cycle 2: 20 cases, cycle 3: 18 cases). Recommendations were informed by multiple verbal comments made by participants during group discussions in each session, with recommendations being uniquely generated for each case. ECHO Autism staff took notes during the session and formulated written recommendations after the session, which were then edited by Hub leads before distribution to participants. There were between 4 and 16 recommendations generated for each of the 62 ECHO cases, with 422 total recommendations forming our data set. The Hub team for each session consisted of the following members: an autistic advocate, one to two family advisors, a developmental pediatrician, a child neurologist, a psychologist, a registered nurse, a board-certified behaviour analyst, an occupational therapist, and a social worker. All Hub members, including autistic advocates and family advisors, were directly involved in the process of discussing and generating the recommendations used in this study.

### Summative approach

We adopted a social constructivist paradigm, acknowledging that as researchers we actively participated in the generation of knowledge (31). This is particularly important given that members of the research team routinely participated in ECHO Autism sessions and have contributed to the generation and writing of recommendations.

A comprehensive content analysis of all Project ECHO Ontario Autism case recommendations was completed using an inductive approach to develop codes and categories of recommendations (32). We employed a summative content analysis approach, in which codes are quantified by calculated frequency counts and interpreted to draw conclusions from the contextual usage of the codes (33).

### Data analysis

Two research team members (one medical student and one ECHO staff member) independently read the recommendations once through and made notes on their first impressions. Next, the two team members developed an original coding guide using recommendations from five ECHO cases that were carefully chosen to ensure a representative sample of cases across all three cycles. The guide was reviewed with a third team member (ECHO Ontario Autism lead and Hub developmental pediatrician) and then was continuously updated during the analytic process. Please see the Supplementary Material for the final version of the coding guide.

Once the initial guide was developed, two research staff independently coded every case and recommendation, meeting regularly to compare and contrast the codes. Any discrepancies were reviewed and resolved with an ECHO lead, including revision of the coding guide and addition of new codes. The team then met to group like codes and identify overarching categories of codes in a hierarchical structure. Finally, the frequencies of each code and category were calculated. Due to the multifaceted nature of the recommendations, a recommendation could be identified with more than one code if it pertained to multiple aspects of autism care. Throughout the entire process, we generated definitions for the categories and codes and kept track of all decisions and changes through analytic memoing. Coding discrepancies between coders that were resolved with the ECHO lead were tracked and used to calculate inter-rater reliability.

## Results

Demographic information about case subjects of the ECHO Autism case presentations are presented in Table 2. Table 2 showcases gender and mean age of all 62 case subjects. Of the 62 cases, 48 (77%) featured male patients, while 14 (23%) featured female patients. Case subject ages ranged from 1.4 years to 21 years. The mean age was 8.5 years, with a standard deviation of 4.6.

**Table 2:** Demographic information of case subjects.

Gender	Number of ECHO case subjects (*n *= 62)
Male	48
Female	14
Gender non-binary, gender non-conforming, or Trans*	0
**Age measurement**	**Age of ECHO case subjects (*n *= 62) in number of years old**
Mean age	8.5
Standard deviation	4.6

Demographic data of ECHO case presentation subjects (*n *= 62), including gender (male, female, gender non-binary, gender non-confirming, or Trans*), and age (from one to twenty-one years of age).

**Figure 2:**
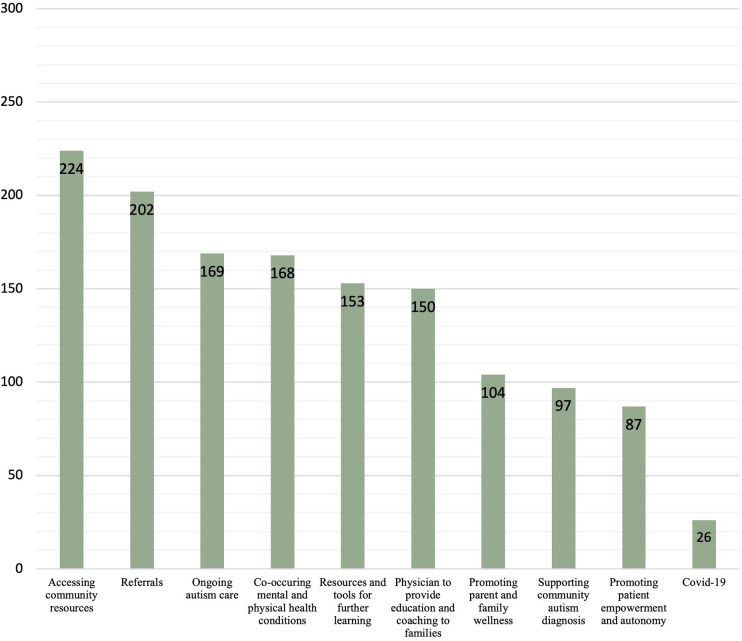
Frequency of categories in recommendations (*n*). Frequency of recommendation categories (*n*) in absolute value of total recommendations (*n *= 422). Categories include: (1) Accessing community resources (*n *= 224), (2) Referrals (*n* = 202), (3) Ongoing autism care (*n* = 169), (4) Co-occurring mental and physical health conditions (*n *= 168), (5) Resources and tools for further learning (*n *= 153), (6) Physician to provide education and coaching to families (*n *= 150), (7) Promoting parent and family wellness (*n *= 104), (8) Supporting community autism diagnosis (*n *= 97), (9) Promoting patient empowerment and autonomy (*n *= 87), and (10) COVID-19 (*n *= 26).

Primary presenting problems of each case were summarized by 3 case codes (Table 3), while case recommendations were described by 55 recommendation codes (Table 4) included in the coding guide, with recommendation codes grouped into ten overarching categories. The three case codes were: (1) diagnosis, (2) management, and (3) both diagnosis and management. Categories of recommendation codes included, in descending order of frequency (*n*/422 total recommendations): (1) accessing community resources (*n* = 224; 53%), (2) referrals (*n* = 202; 48%), (3) ongoing autism care (*n* = 169; 40%), (4) co-occurring mental and physical health conditions (*n* = 168; 40%), (5) resources and tools for further learning (*n* = 153; 36%), (6) physician to provide education and coaching to families (*n* = 150; 35%), (7) promoting parent and family wellness (*n *= 104; 25%), (8) supporting community autism diagnosis (*n* = 97; 23%), (9) promoting patient empowerment and autonomy (*n *= 87; 21%), and (10) COVID-19 (*n* = 26; 6%) (see Figure 2). Of the 1,380 total in-text codes, 68 (4.93%) contained discrepancies that were brought to the ECHO lead for resolution. Therefore, the percent agreement between the two principal coders was 95%.

**Table 3:** Primary presenting problem of ECHO Ontario Autism cases.

Case category	Frequency (*n* = 62)	Percentage	Representative quotation
Diagnosis	16	26%	Cycle 1, Case 1, presenting question: “Does this child have an autism diagnosis?"
Management	31	50%	Cycle 1, Case 4, presenting question: “How else can I help this boy who has increasing behaviours and unpredictable aggression?"
Diagnosis and management	15	24%	Cycle 1, Case 2, presenting question: “Should this boy's autism diagnosis be revisited? How should I address his motor tics and inattention?"

Three case categories [(1) Diagnosis, (2) Management, and (3) Diagnosis and management] were used to code all primary presenting problems of ECHO Autism cases, with each case category frequency (*n*), the percentage out of the total number of cases (*n *= 62), and a representative quotation corresponding to each case category.

**Table 4:** Categories and codes from ECHO Ontario Autism recommendations.

Recommendation category	Codes within category	Code *n*	Recommendation examples
1. Accessing community resources *Total codes = 224; 53% of recommendations (n = 422)*	Accessing funding and navigating resources	53	Cycle 3, Case 12, Recommendation 3: Verify that the family has applied for Developmental Services Ontario (DSO). Mom can apply on her own with a psychological report that states her son has an intellectual disability diagnosis. If he is already receiving DSO, suggest that they access their funded programs such as, respite, behaviour therapy [e.g. at (community agency), see below], recreation, and housing supports. Cycle 2, Case 7, Recommendation 3: Suggest to mom that she seek out sexuality training for her son that will help him to learn age-appropriate rules around touch and hugging. As he is already receiving assistance from the sexual support team at [community agency], they may be able to help support this goal. [Community agency] also offers a sexuality group for teens and their parents.
	Community resources for patient	45	
	School and daycare-based resources	45	
	Community resources for parents and family	39	
	Respite services	16	
	Psychoeducational assessment	15	
	Home-based therapy delivery	11	
2. Referrals *Total codes = 202; 48% of recommendations (n = 422)*	Referral to OT	36	Cycle 3, Case 12, Recommendation 5: Refer this youth to occupational therapy so that mom can receive some sensory strategies to manage his behaviour. Cycle 2, Case 3b, Recommendation 6: Suggest to family that they ask their SLP to focus on the pragmatics of language and social skills. Cycle 3, Case 14, Recommendation 5: Consider talking to the ABA therapist about focusing their intervention on functional communicating using preferred items that he can request instead of matching. Cycle 2, Case 16, Recommendation 4: Consider referring this child for a full autism assessment at [community agency].
	Follow up on referral already made	33	
	Referral to BA/BT	28	
	Referral to other allied health professionals	20	
	Medical specialist referral	19	
	Referral to tertiary care centre or developmental pediatrician outside of Holland Bloorview.	12	
	Referral to SLP	11	
	Referral to Holland Bloorview and the Hub tertiary care centre for resources and programs	10	
	Referral to daycare	9	
	Follow up on referral currently underway	8	
	Future referral suggested	7	
	Referral to Holland Bloorview and the Hub tertiary care centre for specialized physicians and allied health providers	6	
	Referral for participation in a research study	3	
3. Ongoing autism care *Total codes = 169; 40% of recommendations (n = 422*)	Gather more evidence for management	58	Cycle 3, Case 12, Recommendation 4: When a child exhibits a sudden change in behaviour it is important to screen for anything else going on in their life, such as bullying or abuse. Consider having a discussion with parents about any potential stressors in his life and be mindful of possible abuse in the home or community. Cycle 3, Case 12, Recommendation 11: Consider whether this youth's communication system needs a reassessment. Determine whether he is able to communicate pain, his ability to communicate his needs, and whether he has a way to protest things he doesn’t want to do.
	Sensory interventions	24	
	Communication skills and systems	22	
	Daily living skills	21	
	Diet and nutrition	16	
	Sleep interventions	13	
	Physical fitness activities	10	
	Social skills therapy	5	
4. Co-occurring mental and physical health conditions *Total codes = 168; 40% of recommendations (n = 422)*	Co-occurring mental health or developmental diagnosis	66	Cycle 3, Case 12, Recommendation 8: Complete a full medical work up to rule out any organic causes of pain that may be contributing to his behaviour. Include a urine test or ultrasound to investigate whether he has kidney stones that may occur with topiramate. If you need to refer this youth to a more specialized neurologist, [tertiary centre] has a seizure clinic for dual diagnosis (ID and epilepsy).
	Co-occurring physical health diagnosis	37	
	Medical workup	24	
	Mental health supports for patient	22	
	Genetic testing	14	
	Mental health screen	5	
5. Resources and tools for further learning *Total codes = 153; 36% of recommendations* *(n = 422)*	Physician guidance and coaching	57	Cycle 2, Case 3a, Recommendation 2: While this child is on risperidone, order bloodwork following the CAMESA guidelines. Consider ordering a microarray and metabolics, if not completed by the child's neurologist. Please consult the following Canadian Pediatric guidelines for global developmental delays for more guidance on the types of metabolic tests to order. In addition, you may wish to investigate lead and iron, due to this child's pica behaviour. Cycle 3, Case 12, Recommendation 7: You may wish to consider medication to support this youth during this difficult time, as it is currently challenging to access regular programming that would help to decrease this child's interfering behaviours. We suggest discontinuing the olanzapine, since it is not producing the desired effect and is only making him tired. If his psychoeducational assessment and your own assessment supports an ADHD diagnosis, consider targeting his impulsiveness with stimulants or alpha agonists. Alpha agonists can also be helpful for arousal regulation and may therefore be helpful even in the absence of ADHD and much better tolerated than olanzapine.
	Physician educational resources	49	
	Medication guidance and pharmacotherapy	47	
6. Physician to provide education and coaching to families *Total codes = 150; 35% of recommendations (n = 422)*	Parent and family coaching from physician	102	Cycle 1, Case 15a, Recommendation 4: You may choose to provide parents with some suggestions to try to address some of his sensory seeking that may be occurring when he plays with his sister. Suggest that they provide him with him physical input (e.g. hugs or bouncing) before playing with his sister. This may help him better self-regulate when he gets excited during the play. The OT should consider integrating sensory strategies into the plan. Cycle 3, Case 10, Recommendation 11: Consider talking to this youth about the purpose of eating and the function of food as this can be an effective strategy to encourage teens to eat.
	Parent autism education and training	38	
	Patient autism education and training	10	
7. Promoting parent and family wellness *Total codes = 104; 25% of recommendations (n = 422)*	Acknowledging parental and family priorities	32	Cycle 3, Case 4, Recommendation 7: You may consider checking in with his family to assess how they feel about their child's autism diagnosis. If you think they would benefit, contact their local Autism Ontario chapter to connect them to a parent support group.
	Parent and family advocacy	22	
	Acceptance of autism diagnosis	19	
	Safety planning	16	
	Parent and family mental health and wellness	15	
8. Supporting community autism diagnosis *Total codes = 97; 23% of recommendations (n = 422)*	Gather more evidence for diagnosis	49	Cycle 3, Case 2, Recommendation 2: As in this case, it is sometimes more valuable to spend time playing with a child and building a connection than administering a full (formal) assessment. In these scenarios you may wish to administer some items of an ADOS to see what information you can gather. But, as you’ve done, establishing rapport first is essential. Cycle 2, Case 2b, Recommendation 4: Probe to find out what she is doing on her phone in order to determine if this is a repetitive behaviour or if there is a social component.
	Differential diagnosis	32	
	Introduction of autism diagnosis to family	10	
	Meets criteria for diagnosis of autism	6	
9. Promoting patient empowerment and autonomy *Total codes = 87; 21% of recommendations (n = 422)*	Building patient autonomy	30	Cycle 2, Case 14, Recommendation 4: Encourage his family to explore his interests and encourage him to participate in activities where he feels the most competence and confidence. Cycle 2, Case 16, Recommendation 6: Encourage the family to offer more choices in his daily schedule as this may help him gain a sense of control. In addition, try to provide him with high-quality adult attention scheduled throughout the day.
	Building on patient's strengths and interests	19	
	Assisting with transitions	16	
	Social inclusion	14	
	Seeking out trauma-informed care	8	
10. COVID-19 *Total codes = 26; 6% of recommendations (n = 422)*	Impact of COVID-19	26	Cycle 3, Case 12, Recommendation 1: Covid has negatively affected this youth, as his regular programming and supports have been removed and his routine has been disrupted. If possible, connect this youth with services that may provide him with summer programming, such as [community agencies].

Ten recommendation categories [(1) Accessing community resources (*n* = 224), (2) Referrals (*n* = 202), (3) Ongoing autism care (*n* = 169), (4) Co-occurring mental and physical health conditions (*n *= 168), (5) Resources and tools for further learning (*n *= 153), (6) Physician to provide education and coaching to families (*n *= 150), (7) Promoting parent and family wellness (*n *= 104), (8) Supporting community autism diagnosis (*n *= 97), (9) Promoting patient empowerment and autonomy (*n *= 87), and 10) COVID-19 (*n *= 26)] used to code all ECHO Autism cases, with all codes belonging to each category listed, each code frequency (*n*), and representative quotations corresponding to each recommendation category. A recommendation could be identified with more than one code if it pertained to multiple aspects of autism care.

The most common category was **accessing community resources**. Recommendations in this category included parent and sibling support groups, respite services, funding/tax benefits, school and daycare-based resources, requesting a psychoeducational assessment through the school board, and home-based therapy delivery. An example of a representative recommendation in this category is: “Suggest to mom that she seek out sexuality training for her son that will help him to learn age-appropriate rules around touch and hugging. As he is already receiving assistance from the sexual support team at [community agency], they may be able to help support this goal. [Community agency] also offers a sexuality group for teens and their parents.”

The second most common category was **referrals**, which included all references made to referrals to various specialists and allied health providers. Common recommendations were for referrals to be immediately made for speech language pathologists (*n *= 11), occupational therapists (*n *= 36), behavioural therapists (*n *= 28), medical specialists (*n *= 19), and allied health professionals including dieticians, social workers, audiologists, and psychologists (*n *= 20). Please see Table 4 for the complete list of referrals. Recommendations in this category could also include future referrals (*n *= 7), checking in on a referral in-progress (*n *= 8), or noting that the ECHO participant had already made the necessary referral (*n *= 33). One example recommendation suggests: “Refer this youth to occupational therapy so that mom can receive some sensory strategies to manage his behaviour.”

The next category of **ongoing autism care** included building daily living skills such as eating, bathing, and dressing, communication skills and systems, development of social skills, sensory interventions, and prioritizing healthy lifestyle modifications such as physical fitness, nutrition, and sleep habits. An example of a recommendation focusing on communication skills urges participants to “Consider whether this youth's communication system needs a reassessment. Determine whether he is able to communicate pain, his ability to communicate his needs, and whether he has a way to protest things he doesn’t want to do.”

Following this, the next category of recommendations captured diagnosis and management of **co-occurring conditions**. The most common mental health conditions included depression, anxiety, and obsessive-compulsive disorder, while co-occurring neurodevelopmental disorders included attention-deficit/hyperactivity disorder, and a learning or intellectual disability. The most common physical health conditions included feeding and gastrointestinal difficulties, weight-related issues, hearing impairments, and seizures. This category also included recommendations for investigations, such as genetic and medical testing. For example, one recommendation suggested that a clinician “Complete a full medical work up to rule out any organic causes of pain that may be contributing to his behaviour… If you need to refer this youth to a more specialized neurologist, [tertiary centre] has a seizure clinic for dual diagnosis (ID and epilepsy).”

The next category captured **resources and tools for further learning** provided to physician participants. This included recommendations for education programs/materials, such as workshops, training sessions, books, or best practice guidelines. For example, one recommendation advised a clinician that “While this child is on risperidone, order bloodwork following the CAMESA guidelines. Consider ordering a microarray and metabolics, if not completed by the child's neurologist. Please consult the following Canadian Pediatric guidelines for global developmental delays for more guidance on the types of metabolic tests to order.” Recommendations providing education about psychoactive medications were particularly common (*n* = 47).

The following category focused on recommendations asking the **physician to provide education and coaching to families**. Recommendations could be for resources, educational materials, and informal suggestions directed to families and patients to help them better understand autism and self-care strategies. One provider was advised that “You may choose to provide parents with some suggestions to try to address some of his sensory seeking that may be occurring when he plays with his sister. Suggest that they provide him with him physical input (e.g., hugs or bouncing) before playing with his sister.”

The next category centered around **promoting parent and family wellness**, which included a variety of recommendations centering the family's needs and how to best support them. One recommendation suggested that “You may consider checking in with his family to assess how they feel about their child's autism diagnosis. If you think they would benefit, contact their local Autism Ontario chapter to connect them to a parent support group.” Additional recommendations spoke to helping families with pursuing advocacy initiatives, caring for their own mental health, acknowledging parent and family priorities, and family safety planning.

Nearly one quarter of recommendations pertained to **community-based autism diagnosis**, such as encouraging the physician to gather more information from various sources to inform and/or proceed with a possible diagnosis, navigating the differential diagnosis, indicating that the patient meets autism diagnostic criteria, and introducing an autism diagnosis to a child and their family. One community clinician was advised that “… it is sometimes more valuable to spend time playing with a child and building a connection than administering a full (formal) assessment. In these scenarios you may wish to administer some items of an ADOS to see what information you can gather. But, as you’ve done, establishing rapport first is essential.”

A particularly salient category of recommendations spoke to **promoting patient empowerment and autonomy**, with recommendations pertaining to championing social inclusion, assisting with major life transitions, building personal autonomy, acknowledging strengths and interests, and using a trauma-informed care approach. Examples of these recommendations include: “Encourage his family to explore his interests and encourage him to participate in activities where he feels the most competence and confidence” and “Encourage the family to offer more choices in his daily schedule as this may help him gain a sense of control.”

Lastly, a **COVID-19** category was added part way through cycle 2, which corresponded with the start of the COVID-19 pandemic. The COVID-19 category was created to highlight resources which were either created specifically during the pandemic, or were adapted to online service delivery, especially virtual therapy and social activities. This contrasts with general community resources which existed prior to COVID-19 or were not known to be delivered virtually. One youth's experience with COVID-19 was described as follows: “Covid has negatively affected this youth, as his regular programming and supports have been removed and his routine has been disrupted. If possible, connect this youth with services that may provide him with summer programming, such as [community agencies].”

## Discussion

This is the first time that recommendations from ECHO Autism have been characterized and categorized. Our results demonstrate several key areas of learning, including common categories of accessing community resources, making appropriate referrals, and providing ongoing autism care. These categories and their relative frequencies provide important insights into knowledge and care gaps for autistic children and youth.

Our categories are consistent with key areas identified in other studies. Firstly, one study used an originally-developed questionnaire with similar overarching categories to assess self-efficacy of physicians participating in ECHO Autism (25). The questionnaire had five domains, including autism screening and identification, referrals, resources, assessment and treatment of both medical and psychiatric co-occurring conditions, and additional aspects of care for autistic children, all of which are domains replicated in our analysis (25). A Canadian study highlighted several provider-perceived needs for autism identification, including enhanced autism education and greater accessibility of community resources, which were also common categories of recommendations in our study (11). Studies investigating perspectives of Canadian pediatricians on autism diagnosis reported many findings consistent with the ECHO recommendations related to diagnosis, such as strategies for introducing an autism diagnosis to families (34, 35).

Our results reinforce that autism is a multifaceted, heterogeneous condition that often requires input from diversely-skilled professionals. A study investigating Ontario physicians’ perspectives on providing autism care reported that working with interprofessional teams was a facilitator in caring for this population (36). Although interdisciplinary input is ideal, the CPS highlights how access to these resource-intensive teams is scarce and can lead to delays in diagnosis (15). ECHO Autism enables providers to access an interprofessional team despite geographical location, facilitating access to interdisciplinary and specialized knowledge.

Our findings also highlight the importance of access to resources. ECHO participants joined from across Ontario, with presumed varying geographic access to resources. Even still, pediatricians in urban areas expressed difficulties navigating autism resources, which has been previously documented (12). Despite the existence of local autism resources, physicians and families may not be aware of their options, which speaks to the need for effective knowledge sharing of available resources to all stakeholders. Research shows that physicians continue to play a key role in resource dissemination, with parents needing assistance to find reliable autism information (37). A particularly salient finding of our data is a common recommendation for physicians to follow up on referrals that were already made. This points to the increased workload of physicians to be a point of contact between families and providers and spend additional time when communication breaks down in the system of care.

Our findings further demonstrate that clinicians can do more to advocate for autistic children and youth to have more agency and control of their lives, including facilitating social inclusion, building personal autonomy, and acknowledging strengths and interests. Other work has also demonstrated the importance of patient-centered care for autistic patients, including ensuring effective communication, fostering healthy patient-physician relationships, and enabling patient self-management (38). Our findings highlight the importance of training physicians to include family members in autism care; several studies also cite the benefits of shared decision-making, including higher parent satisfaction (39, 40).

Our results have identified post-training gaps in clinical knowledge. Similarly, studies show that autism education remains inadequate in pediatrics residency programs across North America (41). Ontario physicians surveyed about barriers to providing autism care reported a limited focus on autism in medical school and professional development training (36). A study validating objectives of Canadian pediatrics residency programs found that pediatricians reported their preparation for practice was less than adequate in the domain of development and behaviour competencies, including autism (42). Our results suggest that ongoing medical education about autism is needed, as well as guidance about available resources and training to encourage family wellness and prioritize patient empowerment in this population. Based on previously noted comparisons to findings from similar studies across Canada and North America, we believe our results are generalizable to health systems and populations with similar structures to that of Ontario, Canada.

In the international health context, a recent systematic review reported the global prevalence of autism to be 100 per 10,000 individuals (43). While most research into diagnosis and treatment of autism is based on studies in high-income countries such as Canada, similarities between global challenges and our findings exist. For example, one author notes how early detection of autism continues to be a major global challenge, both in high- and low-income countries (44). Furthermore, barriers in access to care including delayed diagnosis, challenges in navigating health systems, and the lack of services and care providers are common issues encountered globally, which are echoed in our results (44). Another similarity stems from available diagnostic and educational services being concentrated in major urban hubs and capital cities, as pointed out in an Ethiopian study (45). Our findings echoed the overrepresentation of ECHO participants and resources from urban areas compared to rural areas. Finally, one study highlights how the innovative use of technology may have the potential to better meet the global needs of autistic individuals and their families (46). ECHO Autism researchers have previously noted the program's flexibility and adaptability to a global context (47).

Our study's key findings point to a greater need for accessibility and awareness of community autism resources and the importance of streamlining allied health referrals, which may be applicable to providers practicing in differently structured healthcare systems than in our study. We believe targeted medical education through programs like ECHO Autism and similar models may benefit practitioners worldwide. ECHO Autism may be further tailored to meet the individual care needs of global communities and systems, such as aiming to increase community autism diagnostic capacity as was the objective with ECHO Ontario Autism, focusing on ongoing management strategies, or a combination of these. Furthermore, engaging in collaborative practice models and resource sharing may help practitioners address our study's recommendations.

Our study was not without limitations. While all ECHO participants had an education level equivalent to that of a medical doctor (MD) or nurse practitioner (NP) practicing in Ontario, ECHO participants may have a higher baseline level of engagement and interest in autism education. Therefore, the cases and knowledge gaps of presenting clinicians may not be representative of all Ontario clinicians. Furthermore, since most participants were based in the GTA, urban perspectives may be over-represented in our findings. Our research team was mostly comprised of ECHO Autism Hub team members and staff, which influenced the interpretation of the data. The data set was composed of summarized key recommendations discussed at each session, which may not represent the diverse set of voices present and capture all individual perspectives that also contributed to learning in the program.

Our results show there is still important work to do in educating clinicians and families to effectively navigate existing autism resources and improve access. Key takeaways include providing continued access to education about autism, streamlining referrals to allied health providers, and having a greater focus on patient autonomy and family well-being.

## Data Availability

The original contributions presented in the study are included in the article/**Supplementary Material**, further inquiries can be directed to the corresponding author.
