# Supplementary material for: Summative content analysis of the recommendations from Project ECHO Ontario Autism
Source: Front Rehabil Sci. 2023 Mar 30;4:1096314. doi: 10.3389/fresc.2023.1096314 (PMC10101203; doi:10.3389/fresc.2023.1096314)
Supplement: Supplementary file 1 [file Table1.docx]

Supplementary Material

# Supplementary Tables

**Supplementary Table 1. Case codes from coding guide**

Three case codes [1) Diagnosis, 2) Management, and 3) Diagnosis and management] used to code all ECHO Autism cases, with accompanying code descriptions.

| Code | Description |
| --- | --- |
| 1. Diagnosis | Code to distinguish a case focusing primarily on a new diagnosis of autism, using diagnostic tools, or collecting more information to make a diagnosis. |
| 2. Management | Code to distinguish a case focusing primarily on management of a pre-existing diagnosis of autism, including medication management, behavioural therapy, and managing concurrent conditions. |
| 3. Diagnosis and management | Code to distinguish a case focusing on both diagnosis and management of autism. |

**Supplementary Table 2. Recommendation codes from coding guide**

55 recommendation codes used to code all ECHO Autism recommendations, with accompanying code descriptions.

| Code | Description |
| --- | --- |
| 1. Gather more evidence for diagnosis | Gathering more information and data for diagnostic purposes, including observational accounts from a variety of sources of home and parent accounts, daycare, and school. Written reports or video recordings may also be used. This also includes information gathered from diagnostic tests and assessments, including the ADOS. |
| 2. Differential diagnosis | Autism is considered as one possible diagnosis as part of a broader differential diagnosis, which may include OCD, ADHD, or other developmental or mental health conditions. |
| 3. Gather more evidence for management | Gathering more information for best management strategies, including evidence of successful strategies from home and school. Gathering more information about potential stressors, including bullying, abuse, and other contextual factors which may affect the implementation of management strategies. |
| 4. Meets criteria for diagnosis of autism | The patient presently meets diagnostic criteria required to proceed with a diagnosis of autism, and the diagnosis is recommended over proceeding with further investigations. |
| 5. Introduction of autism diagnosis to family | Introduce autism diagnosis to parents, caregivers, and patients for the first time. |
| 6. Acceptance of autism diagnosis | Acceptance of autism diagnosis for both patient and parents, provide guidance and emotional support and help with understanding and acceptance of the diagnosis. |
| 7. Co-occurring mental health or developmental diagnosis | Investigating, diagnosing, and/or supporting a child with another developmental or mental health condition in addition to their autism diagnosis, including depression, anxiety, OCD, ADHD, or a learning/intellectual disability. |
| 8. Co-occurring physical health diagnosis | Investigating, diagnosing, and/or supporting a child with another physical health condition in addition to their autism diagnosis, including hearing tests and aids, feeding and gastrointestinal support, seizure/epilepsy management, and obesity and weight-related issues. |
| 9. Medical workup | Recommending completion of a medical workup, which may include bloodwork and labs, an overall medical workup for general health, a specific workup for starting a new medication such as antipsychotics, or following medical guidelines. |
| 10. Genetic testing | Recommending seeking out genetic testing, most commonly a microarray test. |
| 11. Referral to SLP | Referral to a Speech Language Pathologist for speech, language, and communication assessment and therapies. |
| 12. Referral to OT | Referral to an Occupational Therapist for sensory assessments and interventions, recommendations of home and school supports, and assistance with activities of daily living, including behaviours of picky eating. |
| 13. Referral to BA/BT | Referral to a Behavioural Analyst (BA) or Behavioural Therapist (BT) for assessment and management of interfering behaviours. This may include behavioural assessments, functional behavioural analysis, initiating behavioural therapy, and building and creating structured behavioural programs. |
| 14. Referral to other allied health professionals | Referral to another allied health professional, excluding SLP, OT, and BA/BT. This may include a Registered Dietician (RD), Social Worker (SW), Audiologist, or Psychologist for reasons other than psychoeducational assessment or mental health support. |
| 15. Medical specialist referral | Referral to another specialist physician offering expertise of diagnosis or management of complex medical comorbidities. This may include consulting neurology, psychiatry, or a gastroenterologist. |
| 16. Follow-up on referral already made | Provide recommendation for physician to follow up on pre-existing referral already made to various allied health professionals, including SLP, OT, BA/BT, RD, SW, or medical specialists. |
| 17. Follow-up on referral currently underway | Provide recommendation for physician to follow up on a referral that is currently underway but not complete, including to SLP, OT, BA/BT, RD, SW, or medical specialists. |
| 18. Future referral suggested | Provide guidance and recommendation for physician to consider the initiation of a future referral to various allied health professionals, including SLP, OT, BA/BT, RD, SW, or medical specialists. |
| 19. Daily living skills | Advising for the inclusion of skills relating to activities of daily living into the management plan, including eating, dressing, bathing, toileting, pill swallowing, and play. Also includes skills related to technology use for independence in daily life. |
| 20. Communication skills and systems | Advising for the development of communication skills and systems, including referrals to SLP/BA for assessments and communication plans. |
| 21. Social inclusion | Encouraging patient to foster social relationships and inclusion with others and develop connection with peers and members of the community. This may be through community groups, church, or recreation activities that have a social element such as team sports. |
| 22. Social skills therapy | Referral to a dedicated social skills program to enhance child’s social skills and ability to interact with others. |
| 23. Referral to daycare | Referral to a daycare or an early years program so child can develop social skills and socialization in an environment with other peers. |
| 24. Community resources for parents and family | Referral or recommendation of community resources that parents and family members may seek out with the primary goal of providing support to them. This may include parent and caregiver support groups, therapy, and sibling support groups. |
| 25. Respite services | Referral or recommendation that child partakes in respite services to help provide free time for caregivers. |
| 26. Patient autism education and training | Recommendation of various age- and developmentally-appropriate educational resources and reading materials for patients to help them better understand themselves and living with autism. This may include books, videos, educational groups, and other educational sessions. |
| 27. Parent autism education and training | Recommendation of various educational resources and reading materials for parents and caregivers to read to help them better understand their role as a caretaker for an autistic child and management strategies. This may include books, toolkits, and guidelines. This also includes parent skills training groups and other educational sessions. |
| 28. Physician guidance and coaching | Recommendations of guidance and directives on future patient and family interactions, coaching for patient-specific diagnostic and management strategies, and instructions for next steps, future appointments, and follow-up. |
| 29. Physician educational resources | Recommendation of peer education, best practices, frameworks, and educational resources or toolkits for other physicians providing care to the child. |
| 30. Accessing funding and navigating resources | Recommendation of best practices for how parents and families can access funding they are entitled to, including child tax benefits, disability tax credit, Developmental Services Ontario, and Ontario Autism Program. Also includes service navigation programs and helping the family navigate existing resources in their area, including access to therapists and specialists. |
| 31. Community resources for patient | Referral to community groups and programs for patient’s skill building development, which are not already part of a referral to an allied health professional. |
| 32. Mental health supports for patient | Providing recommendations of mental health supports for the patient, including mental health day or inpatient programs, cognitive behavioural therapy, play therapy and assessment, psychology assessment other than psychoeducational assessments, and mindfulness training. |
| 33. Mental health screen | Recommend screening for possible mental health condition, including OCD, anxiety, or depression. |
| 34. School and daycare-based resources | Recommend school-based resources for the child, including psychoeducational assessments through the school, specialized school settings, school-based OT care, and school-based autism teams. Also includes giving recommendations and guidance to teachers and other school-based employees. |
| 35. Psychoeducational assessment | Recommendation that child partake in a psychoeducational assessment or cognitive assessment for possible learning disability and accommodations. This may be done through the school or privately. |
| 36. Parent and family coaching from physician | The physician engages in conversations and coaches parents and caregivers on best practices and evidence-based recommendations for providing optimal care to the child, including establishing home-based routines and communication strategies. This may also include behavioural guidance from the physician and interventions which fall outside of the scope of a referral to a Behavioural Analyst (BA) or Behavioural Therapist (BT). |
| 37. Parent and family advocacy | Provide guidance and support for parents and caregivers to take on an active role in advocating for their child in various settings, including school, daycare, hospital, or other treatment/care centres. |
| 38. Medication guidance and pharmacotherapy | Provide guidance on management strategies of existing prescriptions and regimens, including increasing or decreasing the dosage. Provide recommendations for beginning a new medication prescription and pharmacotherapy as a primary management strategy. |
| 39. Home-based therapy delivery | Recommend allied health professionals and therapy providers come into the patient’s home to provide therapy which is tailored to the home contextual factors and assesses the home environment. |
| 40. Sensory interventions | Recommend sensory interventions and supports for the patient outside of OT referrals, such as noise-cancelling headphones. |
| 41. Physical fitness activities | Recommend the child engages in local sports and physical activities, which may have physical fitness benefits, and may be either individual or team activities. |
| 42. Diet and nutrition | Provide recommendations about diet interventions and changes to increase nutrition and improve eating habits, outside of the scope of a referral to a Registered Dietician. |
| 43. Sleep interventions | Provide recommendations relating to sleep habits, management, the sleep environment, and sleep hygiene. |
| 44. Assisting with transitions | Providing resources and support to help adjust with major life transitions, including transition to daycare, school, moving, or adulthood. |
| 45. Building patient autonomy | Providing recommendations rooted in the principle of patient-centered care, so that the patient may express their concerns and wishes and communicate their needs and desires. Involve patient in the decision-making. Encourage patient to develop skills to improve life-long autonomy beyond medical care. |
| 46. Building on patient’s strengths and interests | Offer recommendations using a strengths-based approach and tailoring activities based on what the child has demonstrated interest and strength in. |
| 47. Acknowledging parental and family priorities | Recognize and address parental priorities with diagnosis and management strategies, and support parents and families with developing a plan that takes into account their priorities while also remaining patient-centered. |
| 48. Parent and family mental health and wellness | Recognize and support parents and family members undergoing mental health concerns, including caregiver-related stress and burnout and pre-existing mental illnesses. Provide guidance on available resources and encourage family members to seek support. |
| 49. Referral to Holland Bloorview and the Hub tertiary care centre for specialized physicians and allied health providers. | Referral to physicians at Holland Bloorview specializing in autism assessment and diagnosis, as well as allied health professionals such as Occupational Therapists and Social Workers accessed through referrals to Holland Bloorview. |
| 50. Referral to Holland Bloorview and the Hub tertiary care centre for resources and programs. | Referral to resources and programs accessed through Holland Bloorview that are not used for diagnostic or assessment purposes. These may include patient, sibling, or parent support groups, therapy or skill building programs, and workshops and educational resources offered through the hospital. |
| 51. Referral for participation in a research study | Referral or recommendation for patient or family members to participate in a research study organized through Holland Bloorview. |
| 52. Referral to tertiary care centre or developmental pediatrician outside of Holland Bloorview. | Referral to resources or supports for complex diagnosis or management accessed through a tertiary care centre other than Holland Bloorview, including accessing developmental pediatricians for diagnosis. |
| 53. Impact of Covid-19 | Case mentions the effects of Covid-19 and the implications of the pandemic on service delivery and the contextual impacts on the child’s functioning, especially online therapy and activities. |
| 54. Seeking out trauma-informed care to address adverse childhood experiences | Case mentions exposure of the child to adverse childhood experiences in the case description. This may include violence, abuse, neglect, or witnessing domestic violence. |
| 55. Safety planning | Recommendation to parents and family to engage in safety planning for child or other siblings in the household. This includes referrals to CAS and other community safety planning organizations. |
